# Prostate Cancer Epigenetics: A Review on Gene Regulation

**DOI:** 10.4137/grsb.s398

**Published:** 2007-12-11

**Authors:** Lena Diaw, Karen Woodson, John W. Gillespie

**Affiliations:** 1 SAIC-Frederick, Inc.- National Cancer Institute/Advanced Technology Center. 8717 Grovemont Circle, Bethesda, Maryland, U.S.A; 2 Cancer Genetics Branch, Center for Cancer Research, National Cancer Institute, National Institutes of Health, Bethesda, Maryland, U.S.A

**Keywords:** epigenetics, methylation, histone modification, gene regulation, prostate cancer

## Abstract

Prostate cancer is the most common cancer in men in western countries, and its incidence is increasing steadily worldwide. Molecular changes including both genetic and epigenetic events underlying the development and progression of this disease are still not well understood. Epigenetic events are involved in gene regulation and occur through different mechanisms such as DNA methylation and histone modifications. Both DNA methylation and histone modifications affect gene regulation and play important roles either independently or by interaction in tumor initiation and progression. This review will discuss the genes associated with epigenetic alterations in prostate cancer progression: their regulation and importance as possible markers for the disease.

## Introduction

Prostate cancer is a major public health problem, as it is the most common cancer diagnosed in men and the leading cause of cancer deaths in the United States. Although recent discoveries in cancer genetics have improved our understanding of prostate carcinogenesis, much remains to be explained concerning the molecular and genetic events important in prostate cancer progression.

Epigenetics is defined as the study of heritable changes in gene expression that are not explained by changes in DNA sequence. Three important mechanisms lead to epigenetic events: DNA methylation, histone modification, and RNA-associated silencing. Among these mechanisms, DNA methylation and histone modification are related to chromatin remodeling have been extensively studied, both often interacting for the control of gene expression (Jones and Baylin, 2002; Santos et al. 2005; Zhang and Dent, 2005). Unlike genetic alterations, which are permanent alterations of DNA sequence, epigenetic changes in tumor and normal cells may have “phenotypic plasticity”. This allows cells to alter their gene expression pattern and adapt to their environment.

In recent years, with the availability of new technologies to further understand the molecular mechanisms in cancer, it has become clear that epigenetic events play a crucial role in cancer (Feinberg and Tycko, 2004). In prostate cancer, it has been shown that DNA methylation and histone modification are important epigenetic mechanisms for changes in gene regulation that can lead to tumorigenesis. These two mechanisms, closely related, sometimes interact to control gene expression (Watanabe et al. 2006; Li and Dahiya, 2007).

In this review, we discuss some of the genes that have been described to frequently be dysregulated in prostate cancer as a consequence of aberrant epigenetic alterations such as DNA methylation and histone modifications.

## Prostate Cancer: Carcinogenesis

In general, prostate cancer has been described as heterogeneous and multifocal, with different clinical and morphological characteristics (Ruijter et al. 1996). Although prostate cancer is generally an indolent disease, 25%–30% of tumors are clinically aggressive (Greenlee et al. 2001; Coffey, 1993).

Prostate cancer is thought to occur initially as an androgen-dependent tumor that, in some cases, can progress to a highly invasive androgen-independent tumor. When the disease is advanced, the tumor spreads locally, metastasizes to the pelvic lymph nodes, and to distant areas like the bone. Once metastasis is initiated, prostate cancer is incurable (Zetter, 1990; Rinker-Schaeffer et al. 1994; Arnold and Isaacs, 2002). During prostate carcinogenesis, multiple cellular and molecular events including genetic changes occur (De Marzo et al. 2003a).

In adults, two main types of prostate disease occur: benign prostatic hyperplasia (BPH) and prostate cancer, which is believed to derive from prostatic intraepithelial neoplasia (PIN) lesions (Untergasser et al. 2005; Chrisofos et al. 2007). Based on their relationship to prostatic disease, three distinct morphological zones have been described in the prostate: (i) the peripheral zone, primarily the site where prostate carcinoma arise; (ii) the transition zone, where BPH mainly occurs; and (iii) the central zone which is relatively resistant to carcinoma and other disease (McNeal, 1969; McNeal, 1988). In prostate cancer development, transformation occurs from benign epithelial glands to pre-malignant lesions and to invasive carcinoma. Some morphological lesions have been proposed as potential precursor of prostate cancer, such as high-grade PIN (Chrisofos et al. 2007) and proliferative inflammatory atrophy (PIA) (De Marzo et al. 1999; De Marzo et al. 2003b).

## Background on Epigenetic Events

Epigenetic mechanisms such as DNA methylation and histone modification play an essential role in many molecular and cellular alterations associated with the development and progression of prostate cancer (Rennie and Nelson, 1999; Li et al. 2005; Schulz and Hatina, 2006). Although, the majority of the epigenetic changes, as discussed below, occur in prostate cancer, some changes have been characterized in BPH and recent data showed a unique set of genes associated with BPH progression (Li and Dahiya, 2007; Prakash et al. 2002). The role of methylation in regulating alterations of gene expression in BPH has not been established, however.

DNA methylation refers to the covalent bounding of a methyl group specifically to the dinucleotide CpG. This is catalyzed by the family of enzymes, the DNA methyltransferases. It is thought that DNA methylation alters chromosome structure and defines regions for transcriptional regulation. Clusters of CpG sites are found dispersed around the genome and are referred to as CpG islands, stretches of DNA ranging from 0.5 to 5 kb with a GC content of at least 50% (Cross and Bird, 1995). These islands are found in the promoter region of about 60% of genes, in exons and introns, and in repetitive elements. Most CpG islands in the promoter regions are unmethylated whereas CpG islands in intronic regions and repetitive sequences are heavily methylated, perhaps to help the cell identify regions for gene transcription.

Two types of DNA methylation alterations have been demonstrated in human cancers. The first refers to global hypomethylation where the genomes of cancer cells show decreased methylation compared to normal cells (Ehrlich, 2002). This hypomethylation is primarily due to the loss of methylation in repetitive elements and other non-transcribed regions of the genome, which results in genomic instability. The second type of methylation alteration in cancer cells is the methylation of CpG islands that lie in promoter regions of tumor suppressor and other regulatory genes that are normally unmethylated. The promoter regions of these genes are inactivated by methylation and their gene expression silenced. This is referred to as gene hypermethylation.

Alterations in DNA methylation often work in concert with changes in chromatin structure modulated through histone modification to silence gene expression. DNA methylation allows for the binding of DNA methylation-specific binding proteins such as MECP2, MBD1, MBD2, which acts to recruit inhibitors and induce histone modification to its inactive state (Watanabe et al. 2006; Li and Dahiya, 2007). In addition, methylation in or near promoter sites may work to directly inhibit the binding of transcription factors to their recognition sequences (Attwood et al. 2002).

## Hypermethylation and Prostate Cancer

**Figure 1:**
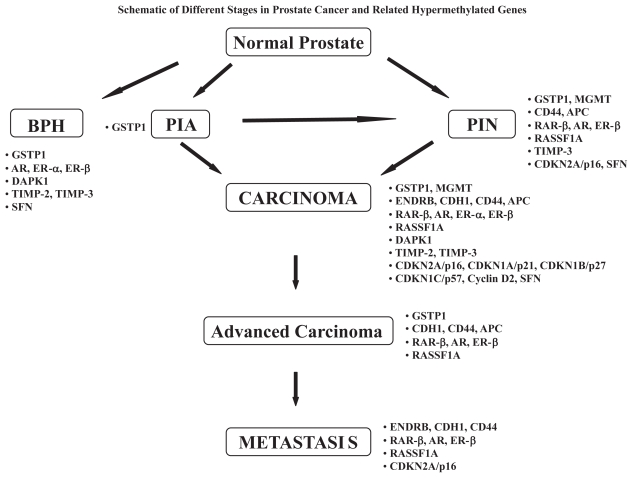
BPH: Benign Prostatic Hyperplasia; PIA: Proliferative Inflammatory Atrophy; PIN: Prostatic Intraepithelial Neoplasia; GSTP1: Glutathione S Transferase Pi; MGMT: O-6-Methylguanine DNA-Methyltransferase; ENDRB: Endothelin Receptor B; CDH1: E-cadherin - CD44; APC: Adenomatous Polyposis Coli; RARβ: Retinoic Acid Receptor beta; AR: Androgen Receptor; ERα: Estrogen Receptor; ERβ: Alpha Estrogen Receptor Beta; RASSF1A: Ras association domain family 1A; DAPK1: Death-Associated Protein Kinase1; TIMP-2: Tissue Inhibitors of Metalloproteinase-2; TIMP-3: Tissue Inhibitors of Metalloproteinase-3; SFN: 14-3-3sigma.

DNA hypermethylation has been the most common and best-characterized epigenetic event in cancer, including prostate cancer. In prostate cancer, a large number of genes have been found hypermethylated. These genes are correlated with pathological grade or clinical stage, and thought to contribute to initiation and progression of the disease. These genes, shown in Table 1 and discussed below, are involved in a variety of cellular pathways such as DNA damage repair (Glutathione S Transferase P1; O^6^-Methylguanine-DNA-Methyltransferase), signal transduction (RASSF1A), adhesion (Endothelin Receptors, E-cadherin, CD44, Adenomatous Polyposis Coli gene, and galectins), hormonal responses (Retinoic Acid Receptor; Androgen Receptor, and Estrogen Receptor), apoptosis (Death-Associated Protein Kinase), cell growth, invasion and metastasis (Tissue Inhibitors of Metalloproteinases, and galectins), and cell cycle control (cyclins, cyclin dependent kinases, and their inhibitors). A schematic depicting the different stages in prostate cancer and related hypermethylated genes is shown in Figure 1.

### Glutathione S transferases

The glutathione S transferases (GSTs) are a family of enzymes involved in intracellular detoxification of xenobiotics and carcinogens by conjugation to glutathione, ultimately protecting cells from DNA damage and cancer initiation (Rushmore and Pickett, 1993; Berhane et al. 1994). Several isoforms of human GST have been described: five cytosolic forms—alpha, mu, pi, sigma, and theta—and one membrane form. Among these genes, pi isoform (GSTP) is the most ubiquitously expressed and well-studied gene (Hayes and Pulford, 1995; Henderson et al. 1998).

In prostate cancer, decrease or loss of GSTP1 expression as a result of gene promoter methylation is the most frequent epigenetic alteration observed (Lee et al. 1994; Maruyama et al. 2002; Yamanaka et al. 2003; Kang et al. 2004; Woodson et al. 2004a; Yegnasubramanian et al. 2004). Silencing of GSTP1 has been shown in 70% to 100% of cancerous lesions, 50%–70% of PIN lesions, whereas it has been rarely detected in normal prostate or BPH tissues (Brooks et al. 1998; Jeronimo et al. 2001; Jeronimo et al. 2002; Yamanaka et al. 2003; Nakayama et al. 2003; Goeman et al. 2003). Recently, GSTP1 gene methylation has been found in a subset of PIA lesions, which are believed to be a precursor for tumors (Nakayama et al. 2003). Also, racial differences have been correlated to GSTP1 methylation status (Woodson et al. 2003; Woodson et al. 2004b).

### O^6^-Methylguanine-DNA-Methyltransferase

The DNA-alkyl repair gene O^6^-Methylguanine-DNA-Methyltransferase (MGMT) is involved in DNA damage repair; it removes mutagenic and cytotoxic alkyl adducts from O^6^-guanine in DNA (Pegg, 1990; Esteller and Herman, 2004). Hypermethylation of the MGMT promoter leads to the loss of its function in various carcinomas (Soejima et al. 2005); however, prostate cancer data are not clear. Indeed, some studies have reported a lack of significant MGMT methylation in prostate tumors (Maruyama et al. 2002; Yamanaka et al. 2003; Yegnasubramanian et al. 2004), whereas others have detected moderate to high levels (Kang et al. 2004; Konishi et al. 2002).

### RASSF1A

RAS proteins have a major function in extra-cellular signals transduction regulating cell growth, survival and differentiation. Many RAS effectors are known as onco-proteins involved in different mechanisms,—apoptosis, contact inhibition, tumor phenotype-, when over-expressed; on the other hand, less is known regarding the effectors acting as tumor suppressor (Vos et al. 2000; Song et al. 2004; Dammann et al. 2000). Among a new family of genes encoding RAS-binding proteins, RAS association domain family 1 gene (RASSF1) has been identified as a tumor suppressor in many carcinomas (Dammann et al. 2005). RASSF1 gene mainly consists of RASSF1A and RASSF1C transcripts that are expressed on two distinct CpG promoters and present in normal human tissues; however, in many cancer, RASSF1A is inactivated and has been correlated to methylation (Dammann et al. 2003).

In prostate cancer, RASSF1A methylation is either frequently detected in tissue samples or not (Kang et al. 2004; Yegnasubramanian et al. 2004; Woodson et al. 2004a; Woodson et al. 2004b; Maruyama et al. 2002; Liu et al. 2002; Kuzmin et al. 2002) and in the latter case it has been a correlated with prostate-specific antigen level or disease stage (Kang et al. 2004; Yegnasubramanian et al. 2004; Woodson et al. 2004a). Furthermore, several findings indicate that RASSF1A methylation occurs at different stages of prostate cancer development as reported in PIN lesions (Kang et al. 2004; Aitchison et al. 2007) and advanced tumors (Kang et al. 2004; Maruyama et al. 2002; Liu et al. 2002).

### Endothelin receptors

The endothelin (ET), a family of peptides, consists of three isotypes that have potent vasoconstructive properties; they are expressed differently in various cells and tissues (Inoue et al. 1989; Rubanyi and Polokoff, 1994). ET-1 isotype is predominant and produced in endothelial cells. It is a growth regulatory peptide involved in cell proliferation (Yanagisawa et al. 1988; Ortega Mateo and de Artinano, 1997). Two receptors ETA and ETB have been identified; they differ from each other, but belong to the same family of heptahelical G-protein-coupled receptors. Both are found in various cells and tissues with different levels of expression (Sakurai et al. 1992; Goto et al. 1996). The receptor A binds highly with equivalent affinity to ET-1 and ET-2 but not to ET-3, whereas receptor B is nonselective and binds with equal affinity to the three isotypes (Sakurai et al. 1992; Goto et al. 1996).

In prostate cancer, ET-1 is produced by primary and metatstatic cells in vivo (Nelson et al. 1995). As prostate cancer progresses, increased expression of ET receptor A is observed, whereas the expression of receptor B is reduced or lost (Gohji et al. 2001; Nelson et al. 1996). Furthermore, the endothelin receptor B gene has been frequently found methylated in prostate cancer samples, and to a lesser frequency in benign samples (Nelson et al. 1997; Pao et al. 2001; Jeronimo et al. 2003; Woodson et al. 2004a; Yegnasubramanian et al. 2004).

### E-cadherin

E-cadherin is a transmembrane glycoprotein, and a member of the cadherin family of cell adhesion molecules that mediates cell-cell adhesion via calcium-dependent interactions (Kemler, 1993; Hirohashi and Kanai, 2003). E-cadherin, which may function as a tumor suppressor gene in invasion and metastasis, has been shown to be decreased or absent in many cancers, and is predictive of poor patient outcome (Oka et al. 1993; Umbas et al. 1994; Richmond et al. 1997). In prostate cancer, decreased expression of E-cadherin, which is related to tumor progression, has been correlated to hypermethylation of the promoter in patients’ samples and human cell lines as well (Kallakury et al. 2001; Maruyama et al. 2002; Woodson et al. 2004b; Yegnasubramanian et al. 2004; Graff et al. 1995; Li et al. 2001).

### CD44

CD44 is a polymorphic cell adhesion molecule that belongs to a family of integral membrane glycoprotein; CD44 plays a role in cell adhesion and cell-matrix interactions as a receptor for hyaluronic acid and osteopontin (Naor et al. 1997; Rudzki and Jothy, 1997; Underhill, 1992; Weber et al. Science. 1996). In prostate cancer, it has been suggested that CD44 may act as a metastasis suppressor gene, and its down-regulation is associated with tumor progression and metastasis (Nagabhushan et al. 1996; Sy et al. 1997; Lou et al. 1999). The exact mechanism of CD44 down-regulation remains elusive; however an epigenetic mechanism, methylation, is clearly involved in this effect. Indeed, a number of studies, both in human samples and cell lines, have shown hypermethylation at the CpG islands in the promoter region of CD44, resulting in the decrease of its expression (Kallakury et al. 1996; Verkaik et al. 1999; Kito et al. 2001; Woodson et al. 2004a; Woodson et al. 2004b).

### Adenomatous polyposis coli

The adenomatous polyposis coli (APC) gene encodes a multifunctional protein that plays a role in Wnt signaling pathway, cell migration, cell adhesion, and mitosis; it is also known to act as a tumor suppressor gene in familial adenomatous polyposis (Fearnhead et al. 2001; Nathke, 1999).

In prostate cancer development, APC hypermethylation has been detected in its earliest stage, in more than 30% of PIN samples, and as the disease progresses the frequency becomes higher (Kang et al. 2004; Maruyama et al. 2002; Yegnasubramanian et al. 2004).

### Galectins

Galectins are a family of animal lectins that act by specifically binding β-galactosides. Presently, fifteen members have been identified; most of them are ubiquitously expressed in various tissues, whereas others are more specific (Leffler et al. 2004; Liu and Rabinovich, 2005). Among the different members, two galectins, 1 and 3 are the most studied. In cancer, galectins play a role in a number of biological functions such as adhesion, proliferation, differentiation, invasion and metastasis. They exert their functions both extra- and intra-cellularly; however, the mechanism by which they regulate these different functions is still unknown (Van Den Brule et al. 2004; Liu and Rabinovich, 2005).

In prostate cancer progression, galectin-3 is mainly studied; and different reports show a down-regulation of this gene (Ellerhorst et al. 1999; Pacis et al. 2000). Galectin-3 is shown to have dual activity, acting as an anti-tumor protein or aid in tumor progression, dependent on its localization in the nucleus or cytoplasm, respectively (Van Den Brule et al. 2000; Califice et al. 2004). Furthermore, very recently, it has been shown for the first time in prostate cancer cell lines that silencing of galectin-3 expression is regulated by promoter hypermethylation (Ahmed et al. 2007).

### Retinoic acid receptorβ

The retinoic acid receptor (RAR) family, which comprises three subtypes (α, β, and γ), belongs to the superfamily of steroid/thyroid hormone receptors (Evans, 1988; Delescluse et al. 1991). Each subtype consists of several isoforms resulting from different promoter usage and alternative splicing. Among these receptors, RARβ2, expressed in most tissues, has been extensively studied in various cancers where it acts as a tumor suppressor gene (Glass et al. 1991; Gudas, 1992; Liu et al. 1996; Ivanova et al. 2002). Loss or down-regulation of RARβ2 expression appears to be both at genetic and epigenetic levels; in the latter it is associated with methylation in the promoter region (Sirchia et al. 2002; Ivanova et al. 2002; Wang et al. 2003).

In prostate cancer, methylation of RARβ2 in the promoter region is frequently detected in primary tumors and very high in hormone-refractory tumors, but not in BPH and normal samples as well (Nakayama et al. 2001; Maruyama et al. 2002; Yamanaka et al. 2003: Woodson et al. 2004a). Furthermore, methylation also occurs at a low level in PIN samples (Jeronimo et al. 2004a). Taken together, it seems that RARβ2 methylation is an early event in prostate cancer, and an indicator of aggressiveness as the disease progresses to late stages (Nakayama et al. 2001; Yamanaka et al. 2003).

### Androgen receptor

Androgens such as testosterone and 5α-dihydrotestosterone are the main steroid hormones in the prostate. These hormones act through the androgen receptor (AR), which belongs to the family of steroid/thyroid nuclear receptors (Heinlein and Chang, 2004). The AR gene expression in prostate cancer progression occurs through different mechanisms including amplification, mutations, and ligand-independent activation (Visakorpi et al. 1995; Taplin et al. 1995; Tilley et al. 1996). Furthermore, androgen independence is a feature of terminal stages in metastatic prostate cancer and the loss of AR expression in those cells appears to be at the transcriptional level (Tilley et al. 1990; Wolf et al. 1993) rather than involving deletion or mutation mechanisms (Dai et al. 1996).

The presence of CpG islands in the AR suggests that this gene might be regulated by methylation. In prostate cancer, a number of studies have indeed shown methylation in the promoter region of the AR leading to its inactivation; however, the frequency of methylation seems to be low (Jarrard et al. 1998; Kinoshita et al. 2000; Nakayama et al. 2000; Sasaki et al. 2002; Yamanaka et al. 2003). In addition, methylation appears to be more prevalent in hormone refractory tumors than in primary tumors (Kinoshita et al. 2000; Nakayama et al. 2000).

### Estrogen receptors

Estrogens are steroid hormones, which are believed to play an important role in prostate carcinogenesis (Carruba et al. 1996; Carruba, 2006). They act through intracellular receptors, which are also effectors involved in proliferation, differentiation, and development of prostate cells (Carruba et al. 1996; Carruba, 2006). The estrogen receptors (ERs) are members of a nuclear receptor superfamily of ligand-activated transcription factors; at present, two receptors, ER-α and ER-β, have been identified, shown to be expressed in a cell and tissue specific manner, and involved in the regulation of the normal function of reproductive tissues (Mosselman et al. 1996; Grandien, 1996). Several studies have reported the presence of both receptors in normal and cancerous prostate tissues, as well as the loss or down-regulation of ER-β during prostate cancer development (Royuela et al. 2001; Horvath et al. 2001; Leav et al. 2001; Bardin et al. 2004).

The epigenetic mechanism, namely methylation in promoter regions of ER-α and ER-β has been associated with decreased or loss of expression of these two genes in prostate cancer. Both receptors are frequently inactivated by CpG methylation in tumor samples and cell lines as well (Li et al. 2000; Lau et al. 2000; Nojima et al. 2001; Sasaki et al. 2002; Yegnasubramanian et al. 2004). However, regarding ER-β, a high frequency of methylation in the promoter region of the gene has been observed at the early stages of the disease, whereas this frequency declined in metastatic tumors (Nojima et al. 2001; Zhu et al. 2004). It has also been reported the methylation of ER-α and ER-β in BPH but to a lesser extent than in prostate cancer tumors (Li et al. 2000; Nojima et al. 2001).

### Death-associated protein kinase

The death-associated protein kinase (DAPK) family is a member of the pro-apoptotic calcium-regulated serine/threonine kinases; it is ubiquitously expressed in tissues, and its inactivation leads to the loss of important apoptotic pathway (Bialik and Kimchi, 2004). Although different mechanisms may affect DAPK inactivation, it has been shown that mainly aberrant methylation is responsible for silencing of this gene. Thus, DAPK has been found methylated in BPH and prostate cancer samples, but not in PIN samples (Maruyama et al. 2002; Yamanaka et al. 2003; Kang et al. 2004; Yegnasubramanian et al. 2004).

### Tissue inhibitors of metalloproteinases

The tissue inhibitors of metalloproteinases (TIMPs) belong to a family of homologous proteins inhibitors that control the activity of matrix metalloproteinases (MMPs) (Gomez et al. 1997). Presently, four members have been identified; they are involved in a number of biological functions such as cell growth, apoptosis, invasion, metastasis and angiogenesis (Fassina et al. 2000; Lambert et al. 2004).

Few reports investigated the possible epigenetic mechanisms underlying the down-regulation of TIMPs in prostate cancer. Methylation patterns have been studied for TIMP-3 and very recently for TIMP-2. TIMP-3 methylation is detected at low levels both in prostate carcinoma and BPH (Yamanaka et al. 2003; Yegnasubramanian et al. 2004; Jeronimo et al. 2004b). Moreover, Jeronimo et al. reported that this event might be age-dependent and zone-dependent (Jeronimo et al. 2004b). Furthermore, a very recent study of prostate cell lines and primary tumors shows that TIMP-2 down-regulation is associated with methylation of the promoter region (Pulukuri et al. 2007).

### Cell cycle genes

A hallmark of tumor cells is their inability of growth control, which is often associated with lack of regulation of the cell cycle. The cell cycle has multiple checkpoints that are controlled by many molecular regulators; often the regulatory molecules affected in cancer are those involved in the control of the G1/S transition of the cell cycle (Peter and Herskowitz, 1994; Biggs and Kraft, 1995; Kamb, 1995). The regulatory proteins involved in the cell cycle include the retinoblastoma protein (RB), cyclins, cyclin dependent kinases (CDKs), and CDK inhibitors (CDKIs), all of which have been implicated in tumor progression (Sherr, 2000). CDKIs have been described as negative regulators of the cell cycle, and subsequently considered as tumor suppressor genes. They consist of two families, the INK4 family and the CIP/KIP (kinase inhibitor protein) family. The INK4 family is composed of four members CDKN2A or p16, CDKN2B or p15, CDKN2C or p18, and CDKN2D or p19, which specifically inhibits CDKs 4 and 6 (Ruas and Peters, 1998; Ortega et al. 2002). The CIP/KIP family includes CDKN1A or p21, CDKN1B or p27, and CDKN1C or p57; they inhibit most CDKs (Sherr and Roberts, 1995). Genetic and/or epigenetic alterations in regulatory molecules and growth pathways directly or indirectly involved in cell cycle control may result in gene inactivation and consequently to deregulation of cell cycle progression, therefore contributing to the pathogenesis of cancer (Macaluso et al. 2005).

In prostate cancer, cell cycle genes can be inactivated by a number of mechanisms such as deletion, point mutation, and hypermethylation. p16 expression has been found up regulated in prostate cancer (Faith et al. 2005). CpG island methylation of p16 gene, which has been observed in prostate cell lines, appears rare in prostate cancer tissues (Herman et al. 1995; Jarrard et al. 1997; Gu et al. 1998; Nguyen et al. 2000; Konishi et al. 2002; Maruyama et al. 2002). Furthermore, in prostate cancer tissues, p16 methylation has been frequently detected at exon2 compared to the promoter region; however, the significance of this event is unclear (Nguyen et al. 2000; Konishi et al. 2002). In addition, cyclin D2 promoter methylation has been detected, and correlated with disease progression in prostate cancer (Padar et al. 2003). Regarding the CIP/KIP family, although epigenetic silencing of p57 is not a rare event, the other members p21 and p27 are rarely methylated in prostate tumors (Konishi et al. 2002; Bott et al. 2005; Kibel et al. 2001; Lodygin et al. 2005a).

The 14-3-3 families of proteins play an important role in regulating cellular signaling involved in cancer development (Hermeking, 2003). Among the different genes in this family, the 14-3-3sigma (SFN) isoform has been mostly implicated in human cancer. It is thought to act as a tumor suppressor gene by inhibiting cell cycle progression (Hermeking et al. 1997; Hermeking, 2003). CpG island methylation and loss of SFN expression have been detected in different types of cancer (Hermeking, 2003; Lodygin and Hermeking, 2005b). SFN, a negative cell cycle regulator, has been found down regulated in prostate cancer cell lines and tissues by promoter hypermethylation (Lodygin and Hermeking, 2005b; Mhawech et al. 2005; Lodygin et al. 2004). SFN methylation has been also observed in BPH tissues (Henrique et al. 2005).

## Hypomethylation and Prostate Cancer

A second type of aberrant methylation, hypomethylation, has also been described to occur in neoplastic cells. This form of DNA methylation occurs principally in many tumors of advanced stages and is thought to be genome-wide (Santos et al. 2005). Hypomethylation or demethylation of normally methylated DNA may lead to structural and functional alterations of the genome. Two types have been described: (i) global or genomic hypomethylation, which is defined as the decrease in overall level of DNA cytosine methylation, and affects different types of repetitive sequences, and (ii) localized or gene-specific hypomethylation, which refers to a decrease in methylation compared to the normal level. Both types have been involved in human cancers (Dunn, 2003).

In general, global and gene hypomethylation in prostate cancer has not been well studied thus far. Global hypomethylation has been observed in a few prostate cancer cases. Prostate cancer cells showed a decrease in overall methylation compared to normal prostate cells (Brothman et al. 2005). Moreover, other studies found associations between global hypomethylation and clinical and metastatic stage of prostate cancer (Schulz et al. 2002; Kindich et al. 2006).

Gene-specific hypomethylation refers to the loss of methylation of gene promoters that are normally methylated. Several studies have found gene hypomethylation in prostate cancers. The following genes affected by this mechanism are urokinase plasminogen activator, cancer/testis antigen gene (CAGE), heparanase, cytochrome P450 1B1 (CYP1B1) and X (inactivate)-specific transcript (XIST) (Table 2). All of these genes have been shown over-expressed and associated with specific stage of the disease (Helenius et al. 2001; Pakneshan et al. 2005; Cho et al. 2002, 2003; Ogishima et al. 2005; Tokizane et al. 2005; Laner et al. 2005). These preliminary studies suggest of gene hypomethylation as a potential mechanism in the up-regulation of genes involved in prostate cancer. This needs to be explored further.

## Histone Modifications and Prostate Cancer

The basic structural unit of DNA, chromatin, is composed of nucleosomes. Nucleosome consists of an octamer of core histones, H2A, H2B, H3 and H4, tightly bound to DNA, and the strength of this interaction is crucial to regulate gene expression (Kornberg and Thomas, 1974a; Kornberg, 1974b; Struhl, 1998; Grunstein, 1997). Five posttranslational modifications of histone proteins, involved in regulation of gene expression, have been identified: acetylation, phosphorylation, methylation, ubiquitination, and ADP-ribosylation; (Ruiz-Carrillo et al. 1975; Jenuwein and Allis, 2001). DNA and histones are linked functionally to control transcription and repair. It has been shown that methylated DNA recruits histone deacetylase (HDAC) through methyl-DNA binding proteins (MBPs); consequently, DNA methylation/histone deacetylation cross talk has been suggested to influence gene silencing (Jones et al. 1998; Nan et al. 1998; Bird, 2002; Goll and Bestor, 2002; Jones and Baylin, 2002; Fahrner et al. 2002; Nguyen et al. 2002).

In prostate cancer, a number of in vitro studies provide evidence that promoter hypermethylation and histone deacetylation interact to maintain chromatin in its inactive state. These studies have shown that combined treatment with the histone deacetylase inhibitor, Trichostatin A, and demethylating agents 5-aza-cytidine or 5-aza-2′-deoxycytidine led to reversing epigenetic silencing of several genes. A loss of hypermethylation in the promoter and concomitant gene activation has been observed for a number of tumor suppressor genes in various prostate cancer cell lines. For example, DAB2IP in PC-3 cell line, RARβ gene in LNCaP, PC-3, and DU145 cell lines, GSTP1 in LNCaP cells, and MAGE a gene that encodes tumor-associated antigens in LNCaP and DU145 cells (Chen et al. 2003; Nakayama et al. 2001; Stirzaker et al. 2004; Wischnewski et al. 2006). These studies provide more evidence for a causative role of DNA hypermethylation and histone modification in the silencing of gene expression.

## Conclusion

In this review, we have described a limited number of genes frequently dysregulated in prostate cancer, postulated to be due to changes in methylation status. Studies show that prostate tumors have a large number of genes with epigenetic changes, indicating that epigenetics plays an important role in the development and progression of prostate cancer. Given this, studies that further knowledge in the epigenetic events related to prostate carcinogenesis may lead to the development of molecular markers for screening and risk assessment, as well as therapeutic targets for preventing and controlling this disease.

## Figures and Tables

**Table 1:** Summary of genes frequently hypermethylated in prostate cancer.

Genes/pathways	Function	Methylation frequency
**DNA damage repair**		
Glutathione S Transferase Pi (GSTP1)	Intracellular detoxification	70% to 100%
O-6-Methylguanine DNA-Methyltransferase (MGMT)	Remove alkyl adducts from O^6^-guanine	0% to 75%
**Cell adhesion**		
Endothelin Receptor B (ENDRB)	Tumor suppressor	38% to 83%
E-cadherin (CDH1)	Tumor suppressor: invasion and metastasis	13% to 70%
**CD44**	Tumor suppressor: metastasis	33% to 68%
Adenomatous Polyposis Coli (APC)	Tumor suppressor	27% to >85%
**Hormonal responses**		
Retinoic Acid Receptor beta (RARβ)	Tumor suppressor	30% to 90%
Androgen Receptor (AR)	Hormone regulation	0% to 28%
Estrogen Receptor Alpha (ERα)	Hormone regulation	19% to 95%
Estrogen Receptor Beta (ERβ)	Hormone regulation	<20% to 100%
**Signal transduction**		
Ras association domain family 1A (RASSF1A)	Tumor suppressor: cell growth	53% to 100%
Death-Associated Protein Kinase1 (DAPK1)	Regulator of cell death	1% to 36%
**Cell growth, invasion, metastasis**		
Tissue Inhibitors of Metalloproteinase-2 (TIMP-2)	Tumor suppressor	78.5%
Tissue Inhibitors of Metalloproteinase-3 (TIMP-3)	Tumor suppressor	6% to 96.6%
**Cell cycle**		
CDKN2A/p16	Tumor suppressor	3% to 66%
CDKN1C/p57	Tumor suppressor	56%
CDKN1A/p21	Tumor suppressor	6%
CDKN1B/p27	Tumor suppressor	6%
Cyclin D2	Cell cycle regulator	32%
14-3-3sigma (SFN)	Cell cycle regulator	40% to 100%

**References:** (GSTP1): Kang, 2004; Woodson, 2004a; Yamanaka, 2003; Yegnasubramanian, 2004. (MGMT): Kang, 2004; Konishi, 2002; Maruyama, 2002; Yamanaka, 2003; Yegnasubramanian, 2004. (ENDRB): Jeronimo, 2003; Nelson, 1997; Woodson, 2004a; Yegnasubramanian, 2004. (CDH1): Li, 2001; Maruyama, 2002; Woodson, 2004b. (CD44): Kito, 2001; Woodson, 2004a, 2004b. (APC): Kang, 2004; Maruyama, 2002; Yegnasubramanian, 2004. (RARβ): Maruyama, 2002; Nakayama, 2001; Yamanaka, 2003; Woodson, 2004a. (AR): Kinoshita, 2000; Nakayama, 2000; Sasaki, 2002; Yamanaka, 2003. (ERα): Li, 2000; Sasaki, 2002; Yegnasubramanian, 2004. (ERβ): Nojima, 2001; Sasaki, 2002; Zhu, 2004. (RASSF1A): Kang, 2004; Kuzmin, 2002; Liu, 2002; Maruyama, 2002; Woodson, 2004a and 2004b; Yegnasubramanian, 2004. (DAPK1): Kang, 2004; Maruyama, 2002; Yamanaka, 2003. (TIMP-2): Pulukuri, 2007. (TIMP-3): Jeronimo, 2004; Yamanaka, 2003. (CDKN2A/p16): Jarrard, 1997; Konishi, 2002; Maruyama, 2002. (CDKN1C/p57): Lodygin, 2005. (CDKN1A/p21): Konishi, 2002. (CDKN1B/p27): Konishi, 2002. (Cyclin D2): Padar, 2003. (SFN): Henrique, 2005; Lodygin, 2004; Mhawech, 2005.

**Table 2:** Summary of hypomethylated genes in prostate cancer.

Genes	Function	Methylation frequency	References
Urokinase Plasminogen Activator (uPA)	Tumor invasion and metastasis	75% to 96.9%	Pakneshan, 2005
Cancer/testis Antigen Gene (CAGE)	Cell cycle control: cellular proliferation	34%	Cho, 2003
Heparanase	Tumor invasion and metastasis	8.5 to 30.5%	Ogishima, 2005
Cytochrome P4501B1 (CYP1B1)	Hydroxylation of estrogens and activation of carcinogens	5.7 to 17.1%	Tokizane, 2005
X (inactivate)-specific transcript (XIST)	X-chromosome inactivation	<4% to >12%	Laner, 2005
